# Claudin-4 polymerizes after a small extracellular claudin-3-like substitution

**DOI:** 10.1016/j.jbc.2024.107693

**Published:** 2024-08-17

**Authors:** Rozemarijn E. van der Veen, Jörg Piontek, Marie Bieck, Arbesa Saiti, Hannes Gonschior, Martin Lehmann

**Affiliations:** 1 Molecular Physiology and Cell Biology, Leibniz-Forschungsinstitut für Molekulare Pharmakologie (FMP), Berlin, Germany; 2 Clinical Physiology/Nutritional Medicine, Department of Gastroenterology, Rheumatology and Infectious Diseases, Charité–Universitätsmedizin Berlin, Berlin, Germany

**Keywords:** tight junction, polymerization, structure-function, claudin, live-STED, confocal microscopy, homology modeling, molecular dynamics

## Abstract

Tight junctions play a pivotal role in the functional integrity of the human body by forming barriers that compartmentalize tissues and protect the body from external threats. Essential components of tight junctions are the transmembrane claudin proteins, which can polymerize into tight junction strands and meshworks. This study delves into the structural determinants of claudin polymerization, using the close homology yet strong difference in polymerization capacity between claudin-3 and claudin-4. Through a combination of sequence alignment and structural modeling, critical residues in the second extracellular segment are pinpointed. Molecular dynamics simulations provide insights into the interactions of and the conformational changes induced by the identified extracellular segment 2 residues. Live-stimulated emission depletion imaging demonstrates that introduction of these residues from claudin-3 into claudin-4 significantly enhances polymerization in nonepithelial cells. In tight junction-deficient epithelial cells, mutated claudin-4 not only influences tight junction morphology but also partially restores barrier function. Understanding the structural basis of claudin polymerization is crucial, as it offers insights into the dynamic nature of tight junctions. This knowledge could be applied to targeted therapeutic interventions, offer insight to repair or prevent barrier defects associated with pathological conditions, or introduce temporary barrier openings during drug delivery.

Barrier formation with the outside world as well as compartmentalization of tissues within the body are essential to keep the human body functional and protected (1). The necessary barriers are formed by epithelial and endothelial cells, and tight junctions (TJs) seal the paracellular space between them (2). While TJs have several cytosolic and transmembrane components, their backbone is formed by claudins (Cldns) (3). The Cldns contain four transmembrane helices, an intracellular N- and C- terminus and two extracellular segments (ECS1 and ECS2) (4). Through a combination of *cis*- and *trans*-interactions they can polymerize into long TJ strands and even more complex meshworks (5, 6). Twenty-five different Cldns have been described in humans (7, 8). Their expression is tissue-specific, where they provide distinct barrier properties (9). Cldn1, for instance, forms a barrier against water and small molecules in the skin. Its loss in Cldn1-deficient mice leads to postnatal death due to dehydration (10). Alternatively, Cldn16 and Cldn19 form a cation-selective pore in the thick ascending limb of the kidney, and their loss causes familial hypomagnesemia with hypercalciuria and nephrocalcinosis (11, 12, 13).

While many years of research have advanced our understanding of the structure and function of TJs, they have often been studied as static structures. Nevertheless, it is crucial to understand their assembly and disassembly as well. Not only is proper TJ assembly important for embryonic pattern formation (14), repair of TJ breaks at cell extrusion sites in the mouse intestine also relies on Cldn polymerization (15). Next to TJ (dis)assembly happening naturally, it can be exploited by pathogens. The C-terminal domain of *Clostridium perfringens* enterotoxin (cCPE) has for instance been shown to disrupt TJ strands through direct interaction with the ECS2 of (mainly) nonjunctional Cldns, blocking their assembly (16, 17, 18, 19). Moreover, *Entamoeba histolytica* cysteine protease (rEhCP112) has been shown to degrade Cldn1 and Cldn2, thereby increasing TJ permeability (20). A better understanding of TJ (dis)assembly could help us prevent or compensate for barrier defects due to these pathogens. Conversely, this knowledge could also be used in drug delivery, where temporary TJ opening could improve delivery efficiency to certain tissues, especially across the very tight blood-brain barrier (21).

Many studies have concentrated on signaling pathways and scaffolding proteins affecting TJ formation (22, 23, 24). However, a more direct approach to modifying TJ (dis)assembly would be to influence Cldn polymerization. Based on the Cldn15 crystal structure (4), models of Cldn15 polymer-based TJ strands have been suggested (6, 25, 26, 27). More recently, polymeric Cldn10a and Cldn10b models have been added to this (28, 29, 30), but models for other Cldns or combinations of Cldns are still missing. From these models, combined with experimental data, it has been proposed that Cldns assemble into *cis*-oligomers before *trans*-interactions at cell-cell contacts trigger polymerization (28, 31, 32). Furthermore, the Cldn ECS2 was found to mediate the *trans*-interaction and thereby drive polymer assembly (28, 33).

Further understanding of Cldn polymerization has been gained from recent imaging advances. Through pulse-chase labeling in epithelial Madin-Darby canine kidney II (MDCKII) cells, it was shown that newly synthesized Cldns are incorporated into the TJ from the basal side (34). Moreover, structured illumination microscopy in living Cldn2-expressing Rat-1 fibroblasts demonstrated additional incorporation at strand break sites (35). Since Furuse *et al.* discovered that Cldn expression can reconstitute TJ strands in fibroblasts (36), these and other nonepithelial cells have been used as a model system to study Cldn polymerization. In a recent study in COS-7 cells, stimulated emission depletion (STED) microscopy revealed that only half of the Cldn family can form polymers on their own (37). Interestingly, in this study Cldn3 polymerized, but its close homolog Cldn4 only gave diffuse membrane staining. Another study in U2OS cells also demonstrated the inability of Cldn4 to form a meshwork (38). Finally, in L-fibroblasts, Cldn3 was found to enrich at the cell-cell contact and form a TJ-like meshwork (5, 16). Cldn4, however, did not show a similar enrichment (16).

In this study we therefore decided to compare Cldn3 and Cldn4 and explore the structural determinants of TJ polymer formation. Given the close homology but strong difference in polymerization capacity of these two proteins, we saw a unique opportunity to understand TJ assembly in more detail. Utilizing sequence alignment and structural modeling, we pinpoint two ECS2 residues that could account for the disparity in polymerization of Cldn3 and Cldn4. Introduction of the corresponding Cldn3 residues in Cldn4 induces meshwork formation, as evidenced by live-STED imaging in COS-7 cells. Moreover, it can partially restore the barrier function and morphology of TJs in TJ-deficient MDCKII cells. We thus discovered an intramolecular motif that shapes the polymerization properties of Cldns.

## Results

### Structural comparison of Cldn3 and Cldn4 indicates residues in ECS2 as critical determinants for polymerization

**Figure 1:**
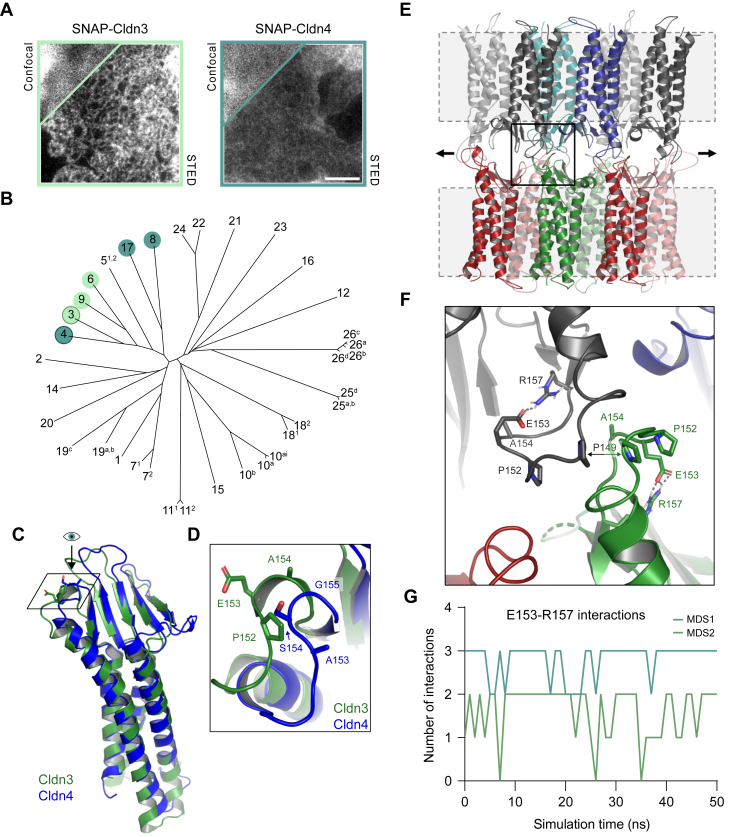
**Sequence alignment and structural investigation of Cldn3 and Cldn4 point toward two residues in ECS2 that may be critical for polymerization.***A*, representative STED and confocal (inset) images of SNAP-Cldn3 and SNAP-Cldn4 in COS-7 cell-cell overlaps. The scale bar represents 1 μm. *B*, phylogenetic tree of the Cldn family, following the nomenclature suggested by Mineta *et al.* (7), demonstrating that meshwork-forming Cldn3, Cldn6, and Cldn9 (in *green*), and nonmeshwork-forming Cldn4, Cldn8, and Cldn17 (in *blue*) fall into the same cluster. *C*, aligned crystal structures of mouse Cldn3 (*green*) and human Cldn4 (*blue*) monomers, derived from cCPE-claudin complexes (PDB 6AKE (58), 7KP4 (59)). *D*, top view of ECS2 region indicated in (*C*), demonstrating key residue differences between Cldn3 (*green*: P152, E153, and A154) and Cldn4 (*blue*: A153, S154, G155) that result in distinct backbone conformations. O atoms are depicted in *red*. *E*, Cldn3 dodecamer model based on previously generated Cldn10b model (29). Snapshot of the MD simulation, showing the overall conformation of all 12 Cldn3 subunits. Cell membranes (not included in simulation) are depicted as *gray dashed boxes*. *Arrows* indicate the direction of strand elongation. *F*, close-up of region outlined in (*E*) showing *trans*-interacting ECS2s of two Cldn3 subunits (*black* and *green*). Intra-molecular electrostatic interactions between E153 and R157 (*dashed lines*; O, *red*; N, *blue*) shape the ECS2 turn together with the rigid P152, allowing the P149 residues of the two subunits to get in close *trans*-proximity. *G*, number of E153-R157 interactions in four central subunits of the Cldn3 dodecamer during two 50 ns MD simulations. cCPE, *Clostridium perfringens* enterotoxin; Cldn, claudin; ECS, extracellular segment; MD, molecular dynamics; PDB, Protein Data Bank; STED, stimulated emission depletion.

This study aims to elucidate structural elements that regulate Cldn polymerization in the TJ and focuses on the close homologs Cldn3 and Cldn4. Using SNAP-tagged human constructs in COS-7 cells we could demonstrate, just as multiple studies did in the past (5, 16, 37, 38), that polymerization of these two Cldns in non-epithelial cells is very distinct: Cldn3 readily forms TJ-like meshworks, whereas Cldn4 does not [Fig. 1*A*]. To determine which residues could underlie this difference, we compared their sequences in a multiple sequence alignment. We further validated amino acid differences by assessing their conservation among meshwork *versus* non-meshwork formers, as classified in COS-7 cells (37). The protein sequences in the Cldn family are widely divergent [Fig. 1*B*] and polymerization modes may differ between members. Therefore, our focus was on the evolutionary most closely related meshwork-forming Cldn6 and Cldn9, and non-meshwork-forming Cldn8 and Cldn17. Cldn5 was excluded, as a past study indicated differences from Cldn3 in an essential domain for *trans*-interaction (39). In the comparison of Cldn3, Cldn6 and Cldn9 *versus* Cldn4, Cldn8 and Cldn17 [Fig. S1], we identified a key difference in ECS2: E153 in Cldn3 *versus* S154 in Cldn4. The larger, negatively charged glutamate in Cldn3 is conserved among Cldn6 (E154) and Cldn9 (E154), whereas Cldn4, Cldn8 (V155) and Cldn17 (I155) all have smaller, polar/hydrophobic residues at this site.

Crystal structures of mouse Cldn3 and Cldn4 (complexed with the C-terminal domain of cCPE) allowed us to examine their ECS2s in more detail [Fig. 1, *C* and *D*]. A comparison showed that Cldn3 and Cldn4 overall have a very similar structure. Nevertheless, the residue identified in our alignment is found in a region with conformational differences, not only caused by this residue (E153/S154 in Cldn3/4), but also the neighboring residues (P152 and A154 in Cldn3; A153 and G155 in Cldn4). When comparing the sequences of all classic Cldns, P152, but not A154, is well conserved in meshwork-formers specifically (3, 37). The proline residue is found in roughly 60% of meshwork forming Cldns and is absent from all Cldns that fail to polymerize. Therefore, P152 and E153 seem of particular interest for meshwork formation. Interestingly, in the Cldn3 as well as the Cldn4 structure, the E153/S154 residue was pointing outwards from the ECS, in the direction that a *trans*-interacting subunit would be positioned (6, 28, 29, 30) [Fig. 1*D*]. This implied the need for a more complex modeling approach to investigate the importance of these residues in polymer formation.

We thus created an oligomeric Cldn3 homology model, based on experimentally derived constraints (28) and a Cldn10b dodecamer template (29). The model was minimized and equilibrated using molecular dynamics (MD) simulations with water as solvent and the transmembrane helices fixed by constraints [Fig. 1*E*]. In strong contrast to its outward facing position in the Cldn3 crystal structure, E153 was found to point inward in the dodecamer model [Fig. 1*F*]. Here, it frequently electrostatically interacts with R157 [Fig. 1*G*], thereby shaping the conformation of the ECS2 turn. This conformation is further stabilized by the neighboring P152 residue that makes the ECS2 turn more rigid and favors close proximity of the P149 residues of two *trans*-facing subunits [Fig. 1*F*]. Based on these simulations, it is thus suggested that the combined rigidity and electrostatic interaction in the ECS2 of Cldn3 (both missing in Cldn4 [Fig. S1],) contribute to a stronger *trans*-interaction and subsequent polymer formation.

### Substituting two Cldn4 residues in the ECS2 with their Cldn3 homologs induces meshwork formation in living nonepithelial cells

**Figure 2:**
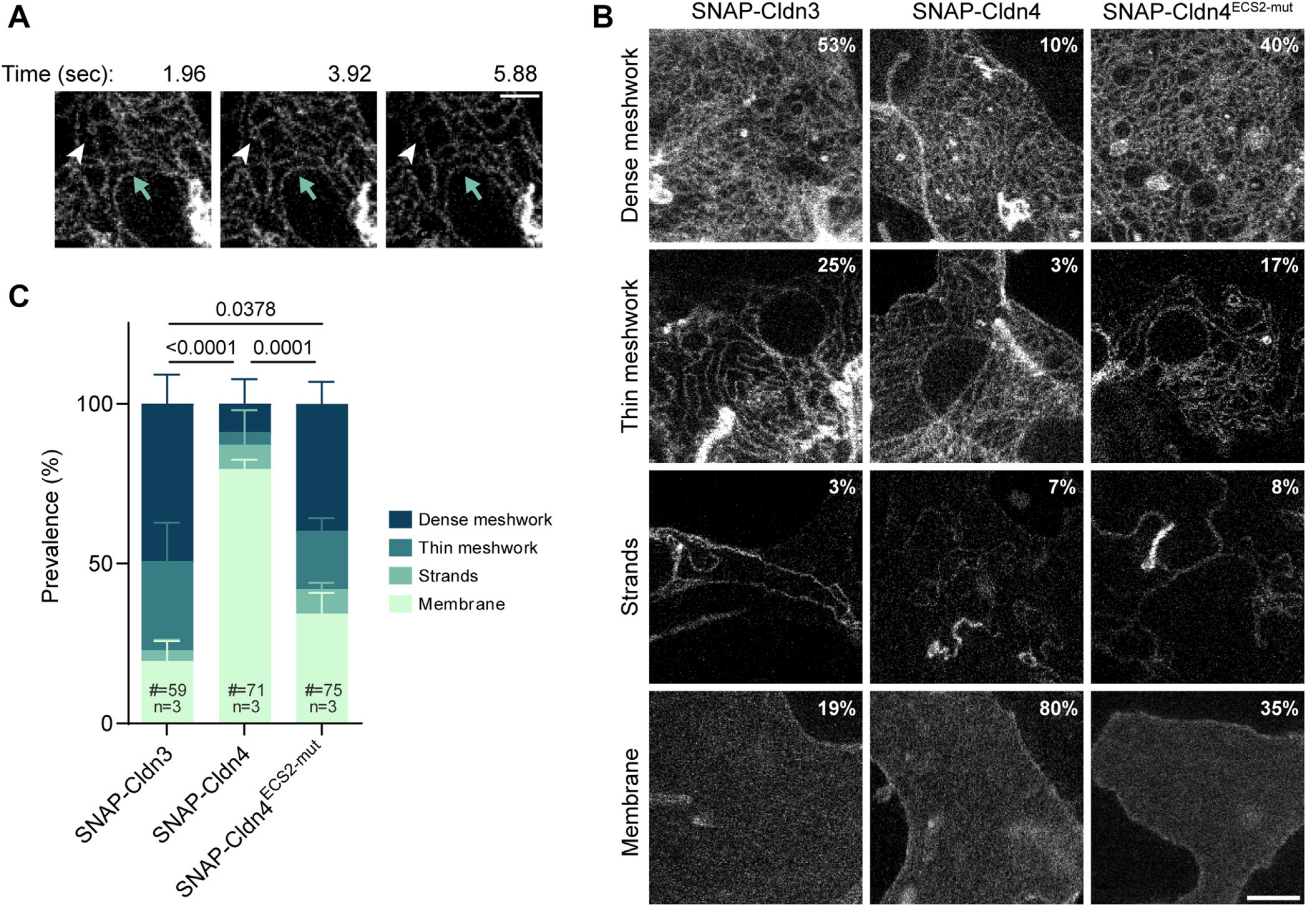
**Systematic STED imaging and classification in living COS-7 cells demonstrates that altering two ECS2 residues of Cldn4 to match those found in Cldn3 can effectively rescue Cldn4's ability to polymerize.***A*, snapshots of a SNAP-Cldn4 meshwork in a COS-7 cell-cell overlap imaged by live-STED, showing upward meshwork movement (*blue arrow*) and strand remodeling (*white arrowhead*). A Gaussian blur of 10 nm was applied to improve image clarity. The scale bar represents 500 nm. *B*, representative images of different structures formed by SNAP-Cldns in COS-7 cell-cell overlaps, along with the corresponding prevalence percentages, pooled from three independent experiments. The scale bar represents 1 μm. *C*, The occurrence of different (non)-polymerized structures in overlaps between COS-7 cells upon SNAP-Cldn expression. Mean + SD from three independent experiments is shown and a one-way ANOVA (*p* < 0.001) with Tukey’s multiple comparison test was performed. Cldn, claudin; ECS, extracellular segment; STED, stimulated emission depletion.

In order to test whether the combined rigidity (of the proline) and negative charge (of the glutamate) are sufficient to promote meshwork formation, we introduced these residues in human Cldn4 (A153P and S154E, from now on denoted as Cldn4^ECS2-mut^). This mutant was analyzed together with human Cldn3 and Cldn4 in COS-7 cells. In order to rule out any artifacts or loss of meshworks due to fixation, living cells were used. In contrast to our previous report using fixated COS-7 cells (37), Cldn4 was found to be occasionally capable of forming meshworks in these living cells [Fig. 2*A*, Video S1]. We analyzed a large amount of cell-cell overlaps and classified whether a dense meshwork, a thin meshwork, isolated strands, or membrane staining was present [Fig. 2, *B* and *C*]. In this way, we could validate that Cldn3 easily polymerizes, forming meshworks 78% of the time, whereas Cldn4 predominantly demonstrates diffuse membrane staining (80%). Interestingly, the two point mutations in the ECS2 of Cldn4 strongly induced polymerization; a meshwork was formed in 57% of the cases.

### Introduction of ECS2-mutated Cldn4 partially rescues TJ morphology and barrier function in epithelial cells

**Figure 3:**
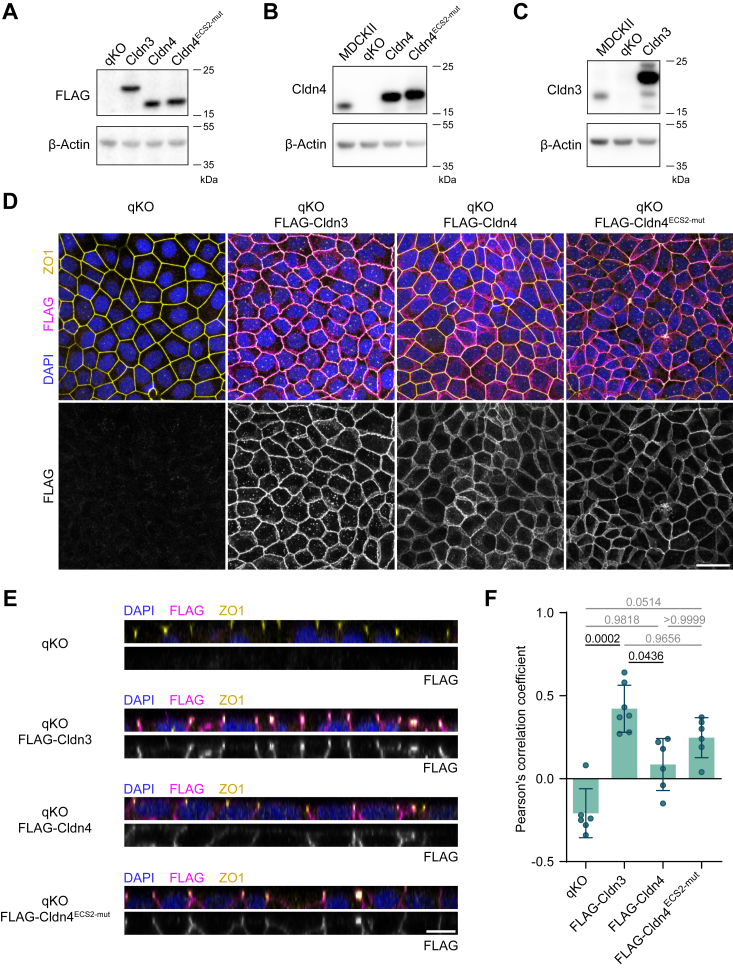
**Establishment of stable Cldn3, Cldn4 and Cldn4**^**ECS2-mut**^**expression in TJ-deficient epithelial cells.***A–C*, stable expression of FLAG-Cldn3, -Cldn4, and -Cldn4^ECS2-mut^ in qKO cells was validated using immunoblotting against the FLAG-tag (*A*), Cldn4 (*B*), Cldn3 (*C*) and β-actin (loading control). *D*, representative maximum intensity projections from an 8 μm z-stack of FLAG-Cldn3, -Cldn4, and -Cldn4^ECS2-mut^ (*magenta*/*gray*) in qKO cells, stained together with the TJ marker ZO1 (*yellow*) and DAPI (*blue*). The scale bar represents 25 μm. *E*, membrane and TJ localization of FLAG-Cldn3, -Cldn4 and -Cldn4^ECS2-mut^ (*magenta*/*gray*) in qKO cells, co-stained with DAPI (*blue*) and ZO1 (*yellow*). Representative orthogonal views of 8 μm z-stacks are shown. The scale bar represents 10 μm. *F*, Colocalization between ZO1 and FLAG-tag in qKO cells (expressing FLAG-Cldn3, -Cldn4 and -Cldn4^ECS2-mut^). Each dot represents one 8 to 9 μm z-stack (n = 6–7, from two independent rounds of immunocytochemistry). Mean ± SD is shown and a Kruskal–Wallis test (*p* ≈ 0.003) with Dunn’s multiple comparison test was performed. Adjusted *p*-values ≤0.05 are shown in *black*, adjusted *p*-values > 0.05 in *gray*. Cldn, claudin; DAPI, 4′,6-diamidino-2-phenylindole; ECS, extracellular segment; qKO, quintuple knockout; TJ, tight junction.

Given the compelling polymerization rescue exhibited in nonepithelial cells, we proceeded to investigate how Cldn4^ECS2-mut^ affects the TJ properties in epithelial cells. For this, we made use of a TJ-deficient MDCK cell line, derived by KO of *Cldn1*, *Cldn2*, *Cldn3*, *Cldn4*, and *Cldn7*, known as claudin quintuple knockout (qKO) cells (40). Using a retroviral expression system, FLAG-tagged Cldn3, Cldn4, and Cldn4^ECS2-mut^ were introduced in qKO cells at similar expression levels [Fig. 3*A*]. All proteins were expressed above their endogenous levels in MDCKII cells [Fig. 3, *B* and *C*] and localized to the plasma membrane [Fig. 3, *D* and *E*]. Except for slight cell to cell variability, protein expression was homogeneous and found in all cells [Fig. 3*D*]. Interestingly, all proteins were found both in the TJ and in the lateral cell membrane [Figs. 3*E* and S2*A*]. When comparing the colocalization with the TJ markers ZO1 and occludin, Cldn3 exhibited the highest, Cldn4 the lowest, and Cldn4^ECS2-mut^ an intermediate degree of overlap [Figs. 3*F* and S2*B*].

**Figure 4:**
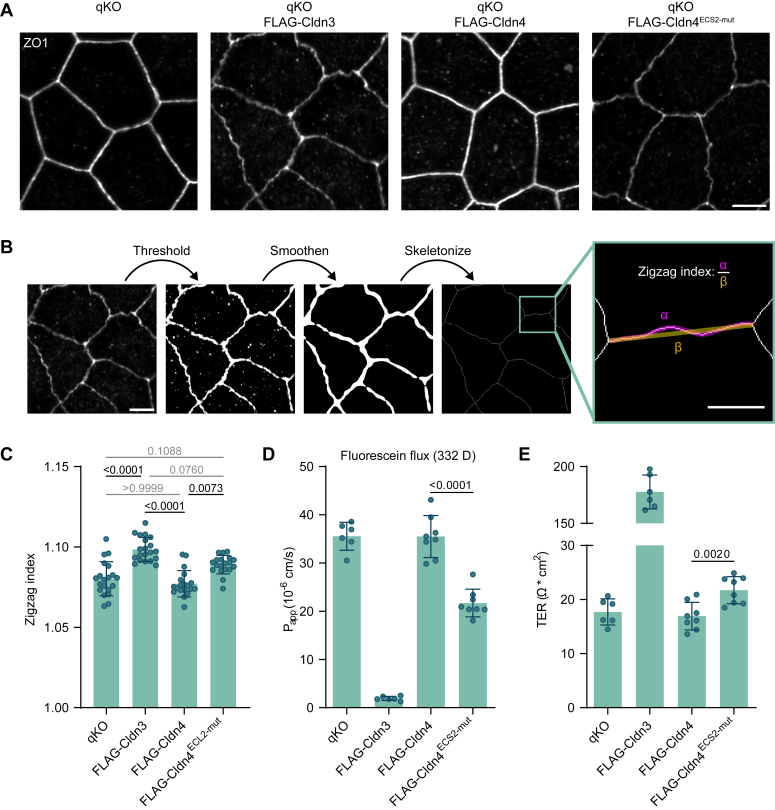
**Cldn4**^**ECS2-mut**^**expression can partially rescue the formation of a ruffled TJ and a barrier against small molecules and electrolytes in qKO cells.***A*, ZO1 staining in qKO cells (expressing FLAG-Cldn3, -Cldn4, or Cldn4^ECS2-mut^), demonstrating distinct TJ morphologies. The scale bar represents 5 μm. *B*, description of the automated pipeline used for quantification of the zigzag index of the TJs. The image of ZO1 in FLAG-Cldn3 expressing qKO cells shown previously in (*A*) was used as an example. On each image the same threshold was applied. Afterward, the images were smoothened and skeletonized. For each cell-cell contact spanning from one tricellular junction to another, the distance covered by the ZO1 signal (α) as well as the shortest distance between the two tricellular junctions (β) was determined. Division of these two resulted in the zigzag index. The scale bars represent 5 μm (main image) and 2 μm (zoom-in). *C*, ZO1 zigzag indexes quantified as described in (*B*) for qKO cells (expressing FLAG-Cldn3, -Cldn4, or -Cldn4^ECS2-mut^). Each dot represents the average zigzag index from one image (24–71 cell-cell junctions were analyzed per image, n = 19–20, from two independent rounds of immunocytochemistry). Mean ± SD is shown and a Kruskal–Wallis test with Dunn’s multiple comparison test was performed. Adjusted *p*-values ≤ 0.05 are shown in *black*, adjusted *p*-values > 0.05 in *gray*. *D*, paracellular permeability for fluorescein (332 D) across qKO cell monolayers (expressing FLAG-Cldn3, -Cldn4, or Cldn4^ECS2-mut^). Every dot represents one transwell filter (n = 6–8). Mean ± SD is shown, and an unpaired two-tailed Student’s *t* test was performed. *E*, evaluation of transepithelial electrical resistance (TER) of qKO cell monolayers (that expressed FLAG-Cldn3, -Cldn4, or -Cldn4^ECS2-mut^). Each dot describes one transwell filter (n = 6–8). Mean ± SD is shown, and an unpaired two-tailed Student’s *t* test was performed. Cldn, claudin; ECS, extracellular segment; qKO, quintuple knockout; TJ, tight junction.

When assessing the ZO1 staining in more detail, clear differences in junctional morphology were observed depending on the expressed Cldn [Fig. 4*A*]. qKO cells display a bright, linear ZO1 signal, as reported before (40, 41), which is unaltered by Cldn4 expression in these cells. Contrastingly, Cldn3 expression led to a more ruffled and dimmer ZO1 signal, similar as observed in MDCKII cells expressing all endogenous Cldns (42). Cldn4^ECS2-mut^ expression also reduced the ZO1 signal and introduced ruffles, though not as strongly as Cldn3. A quantitative measure of junction ruffling is the zigzag index: the ratio between the distance covered by the cell-cell junction from one tricellular contact to the next, and the shortest distance between them. Automatic quantification of the ZO1 zigzag indexes in the different cell types [Fig. 4*B*] gave similar values for qKO cells and cells expressing Cldn4 [Fig. 4*C*], while Cldn3-expressing cells had a significantly higher index. The average index of Cldn4^ECS2-mut^-expressing cells fell in between those of the Cldn3- and Cldn4-expressing cells, but was still significantly different from the latter. These values correlate well with what was found upon *ZO1* KO and reconstitution in MDCKII cells (42): qKO and Cldn4 cell junctions are just as straight as those of *ZO1* KO cells, whereas Cldn3 introduces ruffles comparable to those arising from low-level ZO1 reconstitution. Quantification of the ZO1 intensity at the TJ demonstrated the expected opposing pattern [Fig. S3*A*], with the strongest intensity in qKO cells and Cldn4-expressing cells, the weakest in Cldn3-expressing cells, and Cldn4^ECS2-mut^-expressing cells falling in the middle. There was no apparent difference in total ZO1 expression levels between the cells [Fig. S3, *B* and *C*], either due to the small variance, or regulation occurring exclusively at the level of the TJ.

Finally, we evaluated whether reconstitution of the different Cldns in qKO cells could improve TJ barrier function. Due to the absence of TJ strands, small molecules and electrolytes can easily permeate between qKO cells (40). However, it has been shown that reconstitution of barrier Cldns like Cldn3 can rescue these barrier deficiencies (37, 43). As done in these past studies, we measured the permeability of a small fluorescent tracer molecule (fluorescein, 332 D) [Fig. 4*D*], as well as the transepithelial electrical resistance (TER) [Fig. 4*E*]. We were able to reproduce the barrier deficiency in qKO cells and barrier rescue in Cldn3-expressing cells. Surprisingly, the reintroduction of Cldn4 had no positive impact on barrier function, with no observable changes in fluorescein flux or TER. In contrast, Cldn4^ECS2-mut^ did show the ability to restore barrier function, evident by a marked reduction in fluorescein flux and a slight increase in TER. Overall, we show that Cldn4^ECS2-mut^ can not only rescue polymerization in nonepithelial cells, but also TJ properties in epithelial cells.

## Discussion

In this study, we identified a small motif mediating conformational differences that intrinsically regulate Cldn polymerization. Introduction of only two Cldn3 amino acid residues into the ECS2 of Cldn4 (A153P and S154E) still leaves 19% difference in their protein sequence, yet largely rescues meshwork formation in COS-7 cells. We postulate, based on homology models and MD simulations, that these residues induce an ECS2 conformation favoring *trans*-interaction through rigidity (of the proline) and an intramolecular salt bridge (of the glutamate with R157/158 in Cldn3/4). Given the importance of *trans*-interactions for polymerization (28, 29, 31), this conformational difference can heavily impact polymer formation. When retrovirally introduced into TJ-deficient MDCKII (qKO) cells, it is evident that FLAG-Cldn4 fails to form a barrier for electrolytes and small molecules. Contrary, its mutated counterpart successfully establishes a (partial) barrier and alters junctional morphology from a straight to a ruffled pattern. Additionally, we illustrate that live-STED imaging is an effective tool for identifying morphological distinctions among meshworks formed by various Cldns, thus eliminating potential impacts of fixative agents. This adds to past studies in which super-resolution microscopy was used, either for comparing Cldn meshwork morphology in fixed samples (37) or for analyzing dynamics of only one Cldn (35).

The emphasis of our research was on intrinsic structural properties driving Cldn polymerization, but it should be noted that polymerization is also influenced by context, for example by expression levels. In MDCKII cells that express Cldn4 endogenously, together with Cldn1, Cldn2, Cldn3, and Cldn7, overexpression of Cldn4 increases the TER, decreases the flux of cations and creates a larger TJ meshwork (44, 45). Its KO on the other hand has no apparent effect on the TER or on the ion and small molecule permeability (46). Similar observations were made in opossum kidney cells: overexpression increases the TER, while it is unaffected by knockdown (47). The presence of other Cldns can also make a difference. While Cldn4 in COS-7 cells does not polymerize on its own, it has been shown to copolymerize with Cldn3 and Cldn8 (37). The copolymer with the latter has been suggested to create a chloride channel in the collecting duct (48), which would explain why in collecting duct cell lines, *Cldn4* knockdown does not decrease but increases the TER (48). On the other hand, Cldn4 itself seems to disturb polymerization and thereby inhibit cation channel activity of Cldn2 and Cldn15 (38).

Other factors that could influence Cldn4 polymerization are the different N- or C-terminal tags used in the studies and the species origin of the protein. The importance of all of these factors is especially evident when comparing our data to a recent study by Furuse *et al.* (43). In the same qKO cell line, they find that Cldn4, which did not create a barrier in our experiments, makes TJ strands and introduces a strong barrier against solutes. Despite sharing a similar experimental setup, the two approaches diverge in various aspects. Furuse *et al.* use clones that exhibit a significant overexpression of untagged dog Cldn4, whereas we use a mixed cell population expressing N-terminal FLAG-tagged human Cldn4 at a comparatively lower expression level. It should be noted that the human Cldn4 amino acids that were mutated in this study (A153 and S154) are analogous to the ones in dog Cldn4 (V153 and S154).

While mutating Cldn4 strongly rescued polymerization in COS-7 cells, the effect on the TJ barrier in the qKO cells was smaller. This discrepancy may result from the difference in cell type: COS-7 cells lack the polarity of epithelial cells. Therefore, they may depend less on Cldn trafficking, whereas in the MDCKII-derived qKO cells the proteins need to be correctly targeted to the TJ. This could especially be important for Cldn4, which was shown to have high TJ turnover (compared to Cldn2) in MDCKII cells (34). Upon expression in qKO cells, all Cldns in this study did not only show up in the TJ, but also in the lateral membrane. Endogenous Cldn3/4 and Cldn4 with an N-terminal SNAP-tag have also been shown to localize laterally in MDCKII cells (34). We found TJ localization to be strong for Cldn3, weak for Cldn4, and intermediate for Cldn4^ECS2-mut^.

TJ targeting most likely not only depends on the ECSs, but also on other Cldn domains, in particular the cytosolic C-terminal region. Cldn4 has for instance been shown to depend on its C-terminal region for membrane trafficking (34). The C-terminus of both Cldn3 and Cldn4 has a PDZ motif for binding to the cytosolic adaptor ZO1 (49, 50). Without ZO1 and ZO2, Cldn3 moves from the TJ to the lateral membrane in EpH4 cells (51). Given that Cldn3 and Cldn4 have a vastly different C-terminal region [Fig. S1] that could result in different regulation of ZO binding, their trafficking in epithelial may be very different.

A ruffled junctional morphology as we observed in qKO cells upon expression of only Cldn3 or Cldn4^ECS2-mut^ has been reported in a wide array of articles (41). Usually, ruffle formation is associated with TJ-regulating conditions like hypoxia or anoxia. In these cases, ruffling seems to increase paracellular permeability by increasing TJ circumference (52, 53, 54). This is in stark contrast to our findings, since a loss of ruffling in our system correlates with an increase in paracellular permeability. However, it has been shown that ruffling depends on Cldns (40), ZO1 (42), and their link to the actin cytoskeleton (41, 55), as the loss of any of these leads to a straight, very leaky TJ. We therefore postulate that the combination of a very straight and permeable TJ in our system implies the absence of polymerized Cldns, whereas ruffles combined with TJ barrier formation imply that polymerization has been rescued. In the absence of ruffles, ZO1 intensity at the TJ is also increased. A similar accumulation of ZO1, combined with straightening of the TJ, was observed upon KO of *Cldn1*, *Cldn2*, *Cldn3*, *Cldn4*, and *Cldn7* from MDCKII cells (giving rise to qKO cells) (40). Myosin shows a comparable enrichment in qKO cells (56). ZO1 accumulation may thus be a compensatory mechanism to counteract loss of cell-cell adhesion with enhanced cortical tension.

The Cldn3 polymer model created in this study was based on previously generated Cldn15 and Cldn10b models (6, 29). Recently, another group published computational models for Cldn3 and Cldn4 (57). Nevertheless, their study did not cover several subunit interfaces involved in polymerization, including the ECS2-containing interfaces which were the focus of this study. Our modeling data suggest that for Cldn3 an E153-R157 interaction and the presence of the rigid P152, both absent in Cldn4, shape the ECS2 conformation and thereby strengthen ECS2-ECS2 *trans*-interaction. Of note, the ECS2 conformation and E153 orientation in the simulated Cldn3 oligomers differs from the ones observed in Cldn3 and Cldn4 crystal structures. However, in the latter, the ECS2 conformation is strongly influenced by the binding of the co-crystalized cCPE (58, 59). It is likely that the Cldn ECS2 can adopt different conformations: one stabilized by cCPE binding, the other by Cldn-Cldn *trans*-interaction. Similarly, conformational differences in the TM3/ECS2 of Cldns were reported to affect Cldn-Cldn *cis*-interaction (58).

Interestingly, the electrostatically fitting glutamate-arginine pair is conserved among barrier-forming Cldn3, Cldn6, and Cldn9. Moreover, other barrier-forming claudins have residues at both positions that fit by hydrophobicity (Cldn1, Cldn5, Cldn7, Cldn14, and Cldn19). Channel forming Cldn2, Cldn10b, and Cldn17 are charged only at one of both positions (3) and in Cldn10b channel models, E153 points toward the pore center, where it contributes to cation guidance (29). This covariation suggests that the positions corresponding to E153 and R157 in Cldn3 are in several claudins involved in barrier regulation. At least for R157 in Cldn3 and the corresponding Y158 in Cldn5, involvement in strand formation has been shown previously (33, 39). This is not the first study reporting motifs involved in claudin polymerization (3, 28, 29). However, the previously reported motifs are highly conserved, also between Cldn3 and Cldn4. These could therefore not explain the different polymerization capacity of the two Cldns, in contrast to the novel motif identified in this study. In the future, detailed MD simulations of membrane-embedded oligomers of Cldn3, other barrier-forming claudins and Cldn4 should provide further mechanistic information.

Cldn3 and Cldn4 were the first receptors discovered for CPE, the enterotoxin of *C. perfringens* (60). This bacterial toxin causes the symptoms associated with food poisoning in roughly one million United States citizens each year (61, 62). Even though Cldn3 and Cldn4 are both high affinity CPE receptors, Cldn4 binds the toxin with higher affinity (16). In a study investigating the mechanism of cCPE-Cldn interaction, a similar Cldn3-resembling amino acid substitution in Cldn4 ECS2 (Cldn4 A153P, S154E, G155A) reduced cCPE binding by roughly 22% (63). Modeling and experiments have implied that CPE only binds to nonjunctional Cldns, as TJ Cldns would be sterically inaccessible (17, 18, 19). Thus, we postulate that the identified ECS2 motif not only leads to higher affinity CPE binding of Cldn4 in comparison to Cldn3, but also makes Cldn4 more accessible as a nonjunctional CPE receptor.

In conclusion, we report the identification of a structural determinant of Cldn polymerization and give insight in the (importance of the) ECS2 interface in Cldn polymers. This knowledge can form the basis for further systematic mutational studies and modeling efforts to understand polymer formation across the Cldn family. Ultimately, this could support the design of innovative drugs targeted at precisely modulating the tight junction barrier in different tissues.

## Experimental procedures

### Constructs and molecular cloning

All Cldn sequences used in this study were human. The SNAP-Cldn3 and SNAP-Cldn4 constructs used in this study have been published previously (37). The A153P, S154E mutations in ECS2 of SNAP-Cldn4 were introduced using two primers: 5′-AATCCGCTGGTGCCGGAAGGGCAGAAGC-3′ and 5′-GTAGAAGTCTTGGATGATGTTGTGGGCCGTCCAG-3′ (BioTeZ Berlin-Buch GmbH). Primers were 5′-phosphorylated and site-directed mutagenesis through two-step PCR amplification and ligation was executed according to manufacturer’s protocol (Thermo Scientific Phusion Site-Directed Mutagenesis Kit, F541). Ligation products were used to heat shock transform self-made chemically competent TOP10 *Escherichia coli* cells. Selected colonies were cultured up, and DNA was isolated from them (Macherey-Nagel, 740420.50). Successful creation of SNAP-Cldn4^ECS2-mut^ was verified with sequencing (LGC Genomics GmbH).

pLIB-CMV-FLAG-Cldn4-Puro and pLIB-CMV-FLAG-Cldn4^ECS2-mut^-Puro were created from the previously published pYFP-Cldn4 construct (37), the above described SNAP-Cldn4^ECS2-mut^ plasmid and the published pLIB-CMV-GFP-Puro construct (37). Cldn4 and Cldn4^ECS2-mut^ were PCR amplified and a FLAG-tag was introduced with the following primers: FW 5′-TATAACCGGTATGGATTACAAGGATGACGACGATAAGCTGTACAAAAGCTTGGTACCGAGCTCGGATCCAATGG-3′ and RV 5′-TATAGCGGCCGCGGATTATCTTACACGTAGTTGCTGGC-3′. Two-step PCR amplification was done according to the manufacturer’s protocol (Thermo Scientific Phusion High-Fidelity DNA Polymerase, F530S) and followed up with a PCR clean-up (Macherey-Nagel, F40609.250). PCR products and plasmids were restricted with FastDigest BshTI (Thermo Fisher Scientific, FD1464) and NotI (Thermo Fisher Scientific, FD0593) enzymes. Correct products were isolated with gel electrophoresis and gel extraction (Macherey-Nagel, F40609.250) and were ligated with T4 DNA ligase (Thermo Fisher Scientific, EL0016). HB101 cells (Promega, L2011) were heat shock transformed, DNA was isolated (Macherey-Nagel, 740420.50) and the sequences were checked with sequencing (LGC Genomics GmbH). pLIB-CMV-FLAG-Cldn3-Puro, as well as the pMD2.G retroviral envelope and pCIG3.NB packaging plasmids have been published before (37).

### Cell culture, transient transfection, and SNAP-labeling

In this study, COS-7 (American Type Culture Collection (ATCC) CRL 1651), HEK293T (ATCC CRL 11268), MDCKII (ECACC 00062107) and MDCKII claudin quintuple knockout (qKO, courtesy of Prof. Mikio Furuse, NIPS) cells were used. The cells were regularly tested (negatively) for *mycoplasma*. All the cells were cultured at 37 °C and in a 5% CO_2_ atmosphere in high glucose Dulbecco's modified Eagle's medium (DMEM) (Thermo Fisher Scientific, 11965084), supplemented with 100 μg/ml penicillin-streptomycin (Gibco, Thermo Fisher Scientific, 15140122) and 10% (v/v) fetal bovine serum (FBS) (Gibco, Thermo Fisher Scientific, 10082147). qKO cells stably expressing FLAG-Cldns were maintained with the addition of 2 μg/ml puromycin (Thermo Fisher Scientific, A1113803) in the medium.

For fixed STED imaging, 15.000 cells were seeded per well of a μ-Slide 8-well glass bottom dish (Ibidi, 80827). For live-STED, 200.000 cells were seeded in a glass-bottom 35 mm μ-dish (Ibidi, 81156). Both dishes were coated before with medium containing 2% (v/v) Cultrex Reduced Growth Factor Basement Membrane Extract (Bio-techne, 353600502). Cells were transfected 24 h after seeding using Lipofectamine 2000 Transfection Reagent (Thermo Fisher Scientific, 11668019), according to the manufacturer’s protocol. After this point, cells for fixed imaging were kept in high glucose DMEM with 10% (v/v) FBS, whereas cells for live-STED were kept in FluoroBrite DMEM medium (Thermo Fisher Scientific, A1896701), supplemented with 10% (v/v) FBS. Six hours following transfection the medium containing transfection reagents was replaced by fresh medium. Another 24 h later, the cells were labeled for 1 h at 37  °C and 5% CO_2_ atmosphere with 1 μM (SNAP-ligand) self-labeled BG-JF646, derived from JF646-NHS (Tocris, 6148) and BG-NH_2_ (New England Biolabs Inc, S9148S) (37) in medium. After this, they were washed extensively and incubated for another 30 min in ligand-free medium.

For TJ barrier measurements, 150.000 qKO cells (stably expressing FLAG-Cldns) were seeded on a 12 mm, polycarbonate, 0.4 μm Millicell cell culture insert (Merck, PIHP01250). The medium was refreshed every 2 to 3 days, until cells were confluent and measured 7 days later. On the same day as they were seeded on filters, the qKO cells were seeded in a μ-Slide 8-well glass bottom dish (Ibidi, 80827) for immunofluorescence assays, and in a 6-well plate (Corning, 3516) for immunoblotting. The medium was refreshed every 2 to 3 days. Fixation and lysis were performed 3 to 5 days after, when the cells had reached confluency.

### Creation of stable cell lines

For the production of retrovirus, HEK293T cells were seeded in a 10 cm dish (Sarstedt, 833902), so they would be at roughly 50% confluency 24 h later. The cells were then transfected with the calcium phosphate method: 15 μg of pLIB-CMV-FLAG-Cldn-Puro plasmid was combined with 10.5 μg pCIG3.NB packaging plasmid, 4.5 μg pMD2.G retroviral envelope plasmid, 60 μl 2M CaCl_2_ solution and 440 μl TE buffer (1 mM Tris, 0.2 mM EDTA, pH 8.0), and incubated for 5 min at room temperature (RT). This solution was dropwise added into a 500 μl 2× HBS solution (50 mM Hepes, 280 mM NaCl, 1.5 mM Na_2_HPO_4_, pH 7.05) under slight agitation. After another 20 min of incubation at RT, the resulting solution was used for transfection. The HEK293T medium was refreshed 24 h later. Finally, at 3 to 4 and 5 to 6 days after transfection the medium was spun down for 5 min at 720*g* and virus-containing supernatant was collected.

To generate stable cell lines, qKO cells were seeded so they would be at 50 to 60% confluency 48 h later. The cells were then incubated with 4 ml virus-containing supernatant, filled up to 10 ml with normal medium. After 2 to 3 days of incubation, successfully transduced cells were selected through the addition of 2 μg/ml puromycin to the medium. Selection medium was refreshed every 2 to 3 days, and the cells were split when they were 80 to 90% confluent. After selection, cells were cultured to at least 50% confluency before using them for experiments.

### Fixation and immunocytochemistry

COS-7 cells for fixed STED imaging were fixed for 20 min at 37 °C with prewarmed 4% (w/v) paraformaldehyde and 4% (w/v) sucrose in PBS. qKO cells were fixed with precooled 100% ethanol for 15 min at −20 °C and blocked for 30 min at RT with blocking buffer (6% (v/v) normal goat serum, 1% (w/v) bovine serum albumin, and 0.05% (v/v) Tween-20 in PBS). Primary antibody labeling was done for 2 h at RT in blocking buffer. Rabbit anti-ZO1 antibody (1:100, Thermo Fisher Scientific, 617300), mouse anti-FLAG antibody (1:200, Sigma-Aldrich, F3165), rabbit anti-FLAG antibody (1:200, Sigma-Aldrich, F7425) and mouse anti-Ocln antibody (1:500, Thermo Fisher Scientific, 33-1500) were used. After thorough washing with PBS, secondary antibody labeling in blocking buffer was done for 30 min at RT. For this, goat anti-rabbit Alexa 647 (1:200, Invitrogen, A21244) and goat anti-mouse Alexa 488 (1:200, Invitrogen, A11029) antibodies were used. Finally, the cell nuclei were labeled with blocking buffer containing 4′,6-diamidino-2-phenylindole (DAPI, 1:5000, Thermo Fisher Scientific, 62248), and cells were once more washed thoroughly with PBS.

### STED microscopy

STED imaging was done with a STEDYCON system (Abberior Instruments GmbH, Göttingen), mounted on a Nikon Eclipse TI research microscope, equipped with a Plan APO Lambda 100x/1.4 NA oil objective (Nikon) and controlled by NIS Elements (Nikon). 24 h prior to live-cell imaging, an incubation chamber surrounding the microscope setup was set to 37 °C. In order to provide stable focus during imaging, the Perfect Focus System (Nikon) was used. Imaging was performed in PBS at RT (fixed cells) or in FluoroBrite DMEM medium with 10% (v/v) FBS at 37 °C (living cells). Excitation was done with a 640 nm diode laser, STED depletion with a 775 nm laser. Emission was detected with a single counting avalanche photodiode (650–700 nm). A pixel size of 20 nm by 20 nm and a line accumulation of two for living and 25 for fixed cells were used. For corresponding confocal images, the pixel size was 100 × 100 nm and no line averaging was performed. For time-lapse imaging, the acquisition time was set to 1.96 sec/frame.

### Confocal microscopy

Confocal images of qKO cells were acquired with an LSM780 equipped with spectral detector and PMTs from Carl Zeiss Microscopy. The LSM780 was controlled by the Zeiss Zen Black software (Carl Zeiss Microscopy). For determination of protein localization and colocalization analysis, multi-color confocal imaging was performed in sequential mode with the following fluorophore-specific excitation (Ex.) and emission filter (EmF.) settings: DAPI (Ex.: 405 nm; EmF.: 415–480 nm), Alexa 488 (Ex.: 488 nm; EmF.: 491–561/578 nm), Alexa 647 (Ex.: 633 nm; EmF.: 638–735/752 nm). A PL APO DIC M27 40 x/1.3 NA oil objective, and a zoom factor of 1 were used. Using a line average of 1 to 2, stacks of 8 to 10 images of 512 × 512/1024 × 1024 pixels were acquired at a distance of 1 μm, covering the ZO1/occludin signal in the TJ. For the analysis of TJ ruffles and ZO1 TJ intensity, Alexa 488 (Ex.: 488 nm; EmF.: 491–578nm) and Alexa 647 (Ex.: 633 nm; EmF.: 638–735nm) were sequentially imaged. Images were acquired with a PL APO DIC M27 63 × /1.40 NA oil objective (Carl Zeiss Microscopy) at a zoom factor of 3 and a line average of 2. Stacks of 3 to 6 images (1024 × 1024 pixels) at 1 μm distance were obtained to cover the tight junctional ZO1 in all cells in view.

### Image analysis

Image preparation and analysis was done in Fiji (https://imagej.net/software/fiji/) (64).

#### Meshwork classification

To classify which membrane structures were predominantly formed upon SNAP-Cldn3, -Cldn4, or -Cldn4^ECS2-mut^ expression in living COS-7 cells, all identified cell-cell overlaps were systematically imaged for 1 h per condition. Subsequent classification into membrane staining, isolated strands, thin meshworks, and dense meshworks was done independently by four individuals. Per cell-cell overlap one classifier was assigned, based on the majority vote resulting from the individual classifications.

#### Colocalization analysis

Colocalization, more specifically the Pearson’s correlation coefficient, of the ZO1/occludin channel with the FLAG channel was quantified with the Fiji plugin Coloc 2 (https://imagej.net/plugins/coloc-2). In the case of occludin, 512 × 512 pixel crops were made of the original images. Complete z-stacks were analyzed, using Costes threshold regression, 10 Costes randomizations and a point spread function of 1 pixel (208 nm/415 nm).

#### Automated zigzag index quantification and quantification of ZO1 intensity in the TJ

To quantify the zigzag indexes in the ZO1 channel and the ZO1 levels at the TJ, a maximum intensity projection was performed on the images. After this, the “Triangle Dark” auto threshold was applied, and thresholded images were converted to a mask. The mask was then dilated and smoothened with a median filter (radius: 12 pixels).

For zigzag index quantification, the mask was then skeletonized. The resulting skeleton was analyzed with the Analyze Skeleton (2D/3D) function in Fiji (65). Data were further processed in RStudio (version 4.0.3, https://posit.co/download/rstudio-desktop/). First, any identified branches that were too small to be cell-cell contacts (<2 μm) were eliminated. For all of the remaining branches, now considered cell-cell contacts, the zigzag index was quantified by division of the branch length (α) by the Euclidean distance (β) (Fig. 4*B*). Finally, the mean zigzag index of all cell-cell contacts in the image (ranging from 24 to 71) was calculated.

In the case of ZO1 intensity quantification, any non-TJ structures were deleted from mask by filtering out objects smaller than 4 μm^2^. The mask was then skeletonized, to only select the outline of the TJ. Finally, the whole TJ was covered by reconverting the skeleton to a mask and dilating it 6 times. Within this area, the mean ZO1 intensity was measured. Per experiment (*i.e.* round of immunohistochemistry) the data were normalized to the average TJ ZO1 intensity of all images.

### Cell lysis

Cells were washed once with PBS and lysed with 100 μl ice-cold lysis buffer (1% Triton X-100, 20 mM Hepes, pH 7.4, 130 mM NaCl, 10 mM NaF, and 0.03% protease inhibitor cocktail) on ice. After incubation on ice under constant agitation for 30 min, the lysates were centrifuged at 17,000*g* for 20 min at 4 °C. The supernatant was collected and 1 μl of it was combined with 499 μl H_2_O and 500 μl 2× Bradford reagent. After a 5 min incubation, the protein concentration was determined through measurement of the absorbance at 595 nm with a photometer (BioPhotometer plus, Eppendorf). Finally, 6× SDS sample buffer (0.375 M Tris–HCl (pH 6.8), 10% (w/v) SDS, 60% (v/v) glycerol, 0.6 M DTT, 0.06% (w/v) bromophenol blue) was added, and protein lysates were denatured for 5 min at 95  °C.

### Immunoblotting

Protein size separation of 20 to 30 μg of protein was done on a NuPAGE 4 to 12% Bis-Tris gel (Invitrogen, NP0336BOX) in NuPAGE Mes SDS-buffer (Invitrogen, NP0002) at 100 V for 120 min. The proteins were transferred to a nitrocellulose membrane (Cytiva, 1060004) in transfer buffer (10% (v/v) methanol, 25 mM Tris–HCl (pH 7.6), 192 mM glycin) for 90 min at 110 V on ice. The membrane was blocked with 5% (w/v) milk in TBS-T (0.1% (v/v) Tween-20, 0.01 M Tris-Base, and 0.07 M NaCl, pH 7.6) for 1 h at RT and subsequently incubated with primary antibody in 3% (w/v) bovine serum albumin in TBS-T overnight at 4 °C. Primary antibodies used were mouse anti-FLAG antibody (1:1000, Sigma-Aldrich, F3165), mouse anti-β-actin antibody (1:10,000, Sigma-Aldrich, A5441), rabbit anti-Cldn3 antibody (1:500, Invitrogen, 341700), rabbit anti-Cldn4 antibody (1:500, Invitrogen, 364800) and rabbit anti-ZO1 antibody (1:5000, Thermo Fisher Scientific, 617300). After extensive washing with TBS-T, the membrane was incubated for 1 h at RT with secondary antibody in 5% (w/v) milk in TBS-T. Secondary antibodies used were goat anti-mouse horseradish peroxidase (HRP) (1:5000, Jackson, 115035003) and goat anti-rabbit HRP (1:5000, Jackson, 111035003). After another round of extensive washing with TBS-T and TBS (0.01 M Tris Base, 0.07 M NaCl, pH 7.6), proteins were visualized by the addition of HRP substrate (SuperSignal West Pico PLUS Chemiluminescent Substrate, Thermo Fisher Scientific, 34580) and imaging with a ChemiDoc XRS + imaging system (Bio-Rad) controlled by the Image Lab software (version 6.0.1, https://www.bio-rad.com/de-de/product/image-lab-software).

Immunoblots were quantified in Fiji (64). For each analyzed protein and each immunoblot, consistently sized boxes were drawn around the respective protein band, and the mean intensity was recorded.

### TER and fluorescein permeability measurements

TER and fluorescein flux measurements were done in an Ussing chamber that has been designed to fit the Millicell filters (66). TER was measured with 5 ml of Ringer’s solution (1.2 mM CaCl_2_, 10 mM glucose, 3 mM Hepes, 5.4 mM KCl, 1 mM MgSO_4_, 119 mM NaCl, and 21 mM NaHCO_3_; pH 7.4) on each side. The values were corrected for the resistance of the filter holders and solution on their own. Through constant bubbling (with 95% O_2_ and 5% CO_2_) and heating (to 37  °C), the Ringer’s solution was kept in equilibrium. Before measuring the fluorescein flux, the Ringer’s solution on both sides was increased to 10 ml. After application of a voltage clamp, 10 μl of 100 mM fluorescein was added on the apical side. For 15 min, a small sample was collected in a 96 well plate (Corning, 3365) from the basolateral side every 5 min, and replenished with Ringer’s solution. Fluorescein concentrations were determined with a Tecan Infinite 200 plate reader (Tecan Trading AG) through excitation at 490 nm and detection of emission at 525 nm.

### Sequence alignments

In this article, we mostly adhere to the Cldn nomenclature as suggested by Mineta *et al.* (7) (Table 1). Cldn27, as suggested in this article, was excluded. Another study has demonstrated that this protein is distinct from the Cldn family (8). All Cldn sequences were aligned in Clustal Omega (EBML-EBI) (67) and the outcome was presented as a phylogenetic tree with Drawtree (3.67, phylogeny.fr) (68). For the specific alignment of Cldn3, Cldn4, Cldn6, Cldn8, Cldn9, and Cldn17, PSI/TM-Coffee (version 11.00) was used, with the “transmembrane” and “UniRef100” homology search options (69).

**Table 1:** NCBI reference sequences used in this study

Claudin	Isoform	NCBI reference sequence
1		NP_066924.1
2		NP_065117.1
3		NP_001297.1
4		NP_001296.1
5	1	NP_003268.2
	2	NP_001349995.1
6		NP_067018.2
7	1	NP_001298.3
	2	NP_001171952.1
8		NP_955360.1
9		NP_066192.1
10	a	NP_878268.1
	a_i	NP_001153572.1
	b	NP_008915.1
11	1	NP_005593.2
	2	NP_001171985.1
12		NP_036261.1
14		NP_036262.1
15		NP_055158.1
16		NP_006571.2
17		NP_036263.1
18	1	NP_057453.1
	2	NP_001002026.1
19	a	NP_683763.2
	b	NP_001116867.1
	c	NP_001172046.1
20		NP_001001346.1
21		NP_001094859.1
22		NP_001104789.1
23		NP_919260.2
24		NP_001172078.1
25	a	NP_063948.1
	b	NP_001035272.1
	d	NP_001035290.1
26	a	NP_001139808.1
	b	NP_001277024.1
	c	NP_001277026.1
	d	NP_001277027.1

### Structural modeling and MD simulation

Cldn monomers and oligomers were handled, modeled, simulated, und visualized using the Schrödinger Maestro BioLuminate software platform (BioLuminate, version 4.9.134, Release 2022-4, Schrödinger, LLC, Germany, 2022, https://www.schrodinger.com/platform/products/bioluminate), Schrödinger PyMOL 2.5.2 (http://www.pymol.org/pymol) and Linux-x86_64 GPU computing workstations. Cldn3 homology oligomer models were generated using the murine sequence 1 to 187 (Uniprot ID Q9Z0G9) and the membrane-equilibrated dodecamer model of human Cldn10b (8IBli; (29)) as template. Using the PyMod 3.0.2. plugin for PyMol (http://schubert.bio.uniroma1.it/pymod; (70)), all the 12 Cldn chains/subunits of the Cldn10b dodecamer were replaced by the corresponding Cldn3 homology model. The dodecamer was minimized using the “MacroModel Minimization” module of BioLuminate and a water-solvated environment with a gradient convergence threshold of 0.05 kJ mol^−1^ Å^−1^. This dodecamer model consisted of two *trans*-interacting *cis*-hexamers resulting in three interlocked neighboring *cis*-/*trans*-tetrameric building blocks. Dodecamers were modeled since the middle building blocks are fully connected to the adjacent ones, similar as proposed for polymeric Cldn strands (29). The MD simulations were carried out using the “Desmond Molecular Dynamics” module of BioLuminate (D. E. Shaw Research, New York, NY, 2021. Maestro-Desmond Interoperability Tools, Schrödinger, New York, NY, 2021; (29)). Systems were generated using TIP3P waters, charge-neutralizing ions and 0.15 M NaCl, and the simulations were performed with an OPLS3e force field in NPT ensemble, a temperature of 310 K and a pressure of 1.01325 bar (29). “Desmond Minimization” was performed for 100 ps and the systems were relaxed using BioLuminate default protocol. Afterward, the Cldn3 system was equilibrated stepwise by lowering the constraints on the protein ECS backbone and sidechains from 5 to 0 kcal mol^-1^ Å^-2^ (force constant) over 80 ns while keeping the constraints for the backbone of transmembrane helices on 5 kcal mol^-1^ Å^-2^. After equilibration, the Cldn3 model was simulated with only the transmembrane helices constrained (3 kcal mol^-1^ Å^-2^) for 50 ns (production run MD1). In addition, a stepwise equilibration was performed for 20 ns and followed by simulation with 5 kcal mol^-1^ Å^-2^ only on the backbone of transmembrane helices for 50 ns (production run MD2). Salt interactions between E153 and R157 were analyzed using the MD trajectories and analysis tools within BioLuminate.

### Statistics

Data visualization and statistical analyses were performed in GraphPad Prism (version 9.5.1, https://www.graphpad.com/). Gaussian distribution of the data was verified with multiple normality tests (D’Agostino & Pearson test, Anderson-Darling test and Shapiro–Wilk test).

## Data availability

All data presented in this study are available within the article and the Supporting Information. Further inquiries can be directed to the corresponding authors.

## Supporting information

This article contains supporting information.

## Conflict of interest

The authors declare that they have no conflicts of interest with the contents of this article.

## References

[1] Choi W., Yeruva S., Turner J.R. (2017). Contributions of intestinal epithelial barriers to health and disease. Exp. Cell Res..

[2] Zihni C., Mills C., Matter K., Balda M.S. (2016). Tight junctions: from simple barriers to multifunctional molecular gates. Nat. Rev. Mol. Cell Biol..

[3] Piontek J., Krug S.M., Protze J., Krause G., Fromm M. (2020). Molecular architecture and assembly of the tight junction backbone. Biochim. Biophys. Acta Biomembr..

[4] Suzuki H., Nishizawa T., Tani K., Yamazaki Y., Tamura A., Ishitani R. (2014). Crystal structure of a claudin provides insight into the architecture of tight junctions. Science.

[5] Furuse M., Sasaki H., Tsukita S. (1999). Manner of interaction of heterogeneous claudin species within and between tight junction strands. J. Cell Biol..

[6] Suzuki H., Tani K., Tamura A., Tsukita S., Fujiyoshi Y. (2015). Model for the architecture of claudin-based paracellular ion channels through tight junctions. J. Mol. Biol..

[7] Mineta K., Yamamoto Y., Yamazaki Y., Tanaka H., Tada Y., Saito K. (2011). Predicted expansion of the claudin multigene family. FEBS Lett..

[8] Maher G.J., Hilton E.N., Urquhart J.E., Davidson A.E., Spencer H.L., Black G.C. (2011). The cataract-associated protein TMEM114, and TMEM235, are glycosylated transmembrane proteins that are distinct from claudin family members. FEBS Lett..

[9] Günzel D., Yu A.S.L. (2013). Claudins and the modulation of tight junction permeability. Physiol. Rev..

[10] Furuse M., Hata M., Furuse K., Yoshida Y., Haratake A., Sugitani Y. (2002). Claudin-based tight junctions are crucial for the mammalian epidermal barrier : a lesson from claudin-1–deficient mice. J. Cell Biol..

[11] Simon D.B., Lu Y., Choate K.A., Velazquez H., Al-Sabban E., Praga M. (1999). Paracellin-1, a renal tight junction protein required for paracellular Mg^2+^ resorption. Science.

[12] Konrad M., Schaller A., Seelow D., Pandey A.V., Waldegger S., Lesslauer A. (2006). Mutations in the tight-junction gene claudin 19 (CLDN19) are associated with renal magnesium wasting, renal failure, and severe ocular involvement. Am. J. Hum. Genet..

[13] Hou J., Renigunta A., Konrad M., Gomes A.S., Schneeberger E.E., Paul D.L. (2008). Claudin-16 and claudin-19 interact and form a cation-selective tight junction complex. J. Clin. Invest..

[14] Fleming T.P., Ghassemifar M.R., Sheth B. (2000). Junctional complexes in the early mammalian embryo. Semin. Reprod. Med..

[15] Higashi T., Saito A.C., Fukazawa Y., Furuse M., Higashi A.Y., Ono M. (2022). EpCAM proteolysis and release of complexed claudin-7 repair and maintain the tight junction barrier. J. Cell Biol..

[16] Sonoda N., Furuse M., Sasaki H., Yonemura S., Katahira J., Horiguchi Y. (1999). Clostridium perfringens enterotoxin fragment removes specific claudins from tight junction strands: evidence for direct involvement of claudins in tight junction barrier. J. Cell Biol..

[17] Krause G., Protze J., Piontek J. (2015). Assembly and function of claudins: structure–function relationships based on homology models and crystal structures. Semin. Cell Biol..

[18] Winkler L., Gehring C., Wenzel A., Müller S.L., Piehl C., Krause G. (2009). Molecular determinants of the interaction between Clostridium perfringens enterotoxin fragments and claudin-3. J. Biol. Chem..

[19] Eichner M., Augustin C., Fromm A., Piontek A., Walther W., Bücker R. (2017). In colon epithelia, Clostridium perfringens enterotoxin causes focal leaks by targeting claudins which are apically accessible due to tight junction derangement. J. Infect. Dis..

[20] Cuellar P., Hernández-Nava E., García-Rivera G., Chávez-Munguía B., Schnoor M., Betanzos A. (2017). Entamoeba histolytica EhCP112 dislocates and degrades claudin-1 and claudin-2 at tight junctions of the intestinal epithelium. Front. Cell. Infect. Microbiol..

[21] Dong X. (2018). Current strategies for brain drug delivery. Theranostics.

[22] Stephenson R.E., Higashi T., Erofeev I.S., Arnold T.R., Leda M., Goryachev A.B. (2019). Rho flares repair local tight junction leaks. Dev. Cell.

[23] Beutel O., Maraspini R., Pombo-García K., Martin-Lemaitre C., Honigmann A. (2019). Phase separation of zonula occludens proteins drives formation of tight junctions. Cell.

[24] González-Mariscal L., Tapia R., Chamorro D. (2008). Crosstalk of tight junction components with signaling pathways. Biochim. Biophys. Acta Biomembr..

[25] Alberini G., Benfenati F., Maragliano L. (2017). A refined model of claudin-15 tight junction paracellular architecture by molecular dynamics simulations. PLOS One.

[26] Samanta P., Wang Y., Fuladi S., Zou J., Li Y., Shen L. (2018). Molecular determination of claudin-15 organization and channel selectivity. J. Gen. Physiol..

[27] Fuladi S., McGuinness S., Shen L., Weber C.R., Khalili-Araghi F. (2022). Molecular mechanism of claudin-15 strand flexibility: a computational study. J. Gen. Physiol..

[28] Hempel C., Protze J., Altun E., Riebe B., Piontek A., Fromm A. (2020). Assembly of tight junction strands: claudin-10b and claudin-3 form homo-tetrameric building blocks that polymerise in a channel-independent manner. J. Mol. Biol..

[29] Nagarajan S.K., Klein S., Fadakar B.S., Piontek J. (2023). Claudin-10b cation channels in tight junction strands: octameric-interlocked pore barrels constitute paracellular channels with low water permeability. Comput. Struct. Biotechnol. J..

[30] Nagarajan S.K., Piontek J. (2024). Molecular dynamics simulations of claudin-10a and -10b ion channels: with similar architecture, different pore linings determine the opposite charge selectivity. Int. J. Mol. Sci..

[31] Koval M. (2013). Differential pathways of claudin oligomerization and integration into tight junctions. Tissue Barriers.

[32] Piontek J., Fritzsche S., Cording J., Richter S., Hartwig J., Walter M. (2011). Elucidating the principles of the molecular organization of heteropolymeric tight junction strands. Cell. Mol. Life Sci..

[33] Piontek J., Winkler L., Wolburg H., Müller S.L., Zuleger N., Piehl C. (2008). Formation of tight junction: determinants of homophilic interaction between classic claudins. FASEB J..

[34] Van Itallie C.M., Lidman K.F., Tietgens A.J., Anderson J.M. (2019). Newly synthesized claudins but not occludin are added to the basal side of the tight junction. Mol. Biol. Cell.

[35] Van Itallie C.M., Tietgens A.J., Anderson J.M. (2017). Visualizing the dynamic coupling of claudin strands to the actin cytoskeleton through ZO-1. Mol. Biol. Cell.

[36] Furuse M., Sasaki H., Fujimoto K., Tsukita S. (1998). A single gene product, claudin-1 or -2, reconstitutes tight junction strands and recruits occludin in fibroblasts. J. Cell Biol..

[37] Gonschior H., Schmied C., Van der Veen R.E., Eichhorst J., Himmerkus N., Piontek J. (2022). Nanoscale segregation of channel and barrier claudins enables paracellular ion flux. Nat. Comm..

[38] Shashikanth N., France M.M., Xiao R., Haest X., Rizzo H.E., Yeste J. (2022). Tight junction channel regulation by interclaudin interference. Nat. Comm..

[39] Rossa J., Ploeger C., Vorreiter F., Saleh T., Protze J., Günzel D. (2014). Claudin-3 and claudin-5 protein folding and assembly into the tight junction are controlled by non-conserved residues in the transmembrane 3 (TM3) and extracellular loop 2 (ECL2) segments. J. Biol. Chem..

[40] Otani T., Nguyen T.P., Tokuda S., Sugihara K., Sugawara T., Furuse K. (2019). Claudins and JAM-A coordinately regulate tight junction formation and epithelial polarity. J. Cell Biol..

[41] Lynn K.S., Peterson R.J., Koval M. (2020). Ruffles and spikes: control of tight junction morphology and permeability by claudins. Biochim. Biophys. Acta Biomembr..

[42] Tokuda S., Higashi T., Furuse M. (2014). ZO-1 knockout by TALEN-mediated gene targeting in MDCK cells: involvement of ZO-1 in the regulation of cytoskeleton and cell shape. PLOS One.

[43] Furuse M., Nakatsu D., Hempstock W., Sugioka S., Ishizuka N., Furuse K. (2023). Reconstitution of functional tight junctions with individual claudin subtypes in epithelial cells. Cell Struct. Funct..

[44] Van Itallie C., Rahner C., Anderson J.M. (2001). Regulated expression of claudin-4 decreases paracellular conductance through a selective decrease in sodium permeability. J. Clin. Invest..

[45] Itallie C.M.V., Fanning A.S., Anderson J.M. (2003). Reversal of charge selectivity in cation or anion-selective epithelial lines by expression of different claudins. Am. J. Physiol. Ren. Physiol..

[46] Tokuda S., Hirai T., Furuse M. (2017). Claudin-4 knockout by TALEN-mediated gene targeting in MDCK cells: claudin-4 is dispensable for the permeability properties of tight junctions in wild-type MDCK cells. PLOS One.

[47] Borovac J., Barker R.S., Rievaj J., Rasmussen A., Pan W., Wevrick R. (2012). Claudin-4 forms a paracellular barrier, revealing the interdependence of claudin expression in the loose epithelial cell culture model opossum kidney cells. Am. J. Physiol. Cell Physiol..

[48] Hou J., Renigunta A., Yang J., Waldegger S. (2010). Claudin-4 forms paracellular chloride channel in the kidney and requires claudin-8 for tight junction localization. Proc. Natl. Acad. Sci. USA.

[49] Stiffler M.A., Chen J.R., Grantcharova V.P., Lei Y., Fuchs D., Allen J.E. (2007). PDZ domain binding selectivity is optimized across the mouse proteome. Science.

[50] Itoh M., Furuse M., Morita K., Kubota K., Saitou M., Tsukita S. (1999). Direct binding of three tight junction-associated MAGUKs, ZO-1, ZO-2, and ZO-3, with the COOH termini of claudins. J. Cell Biol..

[51] Umeda K., Ikenouchi J., Katahira-Tayama S., Furuse K., Sasaki H., Nakayama M. (2006). ZO-1 and ZO-2 independently determine where claudins are polymerized in tight-junction strand formation. Cell.

[52] González-Mariscal L., Avila A., Betanzos A. (2001). The relationship between structure and function of tight junctions. Tight Junctions.

[53] Saeedi B.J., Kao D.J., Kitzenberg D.A., Dobrinskikh E., Schwisow K.D., Masterson J.C. (2015). HIF-dependent regulation of claudin-1 is central to intestinal epithelial tight junction integrity. Mol. Biol. Cell.

[54] Jin Y., Blikslager A.T. (2016). Myosin light chain kinase mediates intestinal barrier dysfunction via occludin endocytosis during anoxia/reoxygenation injury. Am. J. Physiol. Cell Physiol..

[55] Fanning A.S., Little B.P., Rahner C., Utepbergenov D., Walther Z., Anderson J.M. (2007). The unique-5 and-6 motifs of ZO-1 regulate tight junction strand localization and scaffolding properties. Mol. Biol. Cell.

[56] Mukenhirn M., Wang C.-H., Guyomar T., Bovyn M.J., Staddon M.F., van der Veen R.E. (2024). Tight junctions control lumen morphology via hydrostatic pressure and junctional tension. Dev. Cell.

[57] Raya-Sandino A., Lozada-Soto K.M., Rajagopal N., Garcia-Hernandez V., Luissint A.-C., Brazil J.C. (2023). Claudin-23 reshapes epithelial tight junction architecture to regulate barrier function. Nat. Comm..

[58] Nakamura S., Irie K., Tanaka H., Nishikawa K., Suzuki H., Saitoh Y. (2019). Morphologic determinant of tight junctions revealed by claudin-3 structures. Nat. Comm..

[59] Vecchio A.J., Rathnayake S.S., Stroud R.M. (2021). Structural basis for Clostridium perfringens enterotoxin targeting of claudins at tight junctions in mammalian gut. Proc. Natl. Acad. Sci. USA.

[60] Katahira J., Sugiyama H., Inoue N., Horiguchi Y., Matsuda M., Sugimoto N. (1997). Clostridium perfringens enterotoxin utilizes two structurally related membrane proteins as functional receptors in vivo. J. Biol. Chem..

[61] Scallan E., Hoekstra R.M., Angulo F.J., Tauxe R.V., Widdowson M.A., Roy S.L. (2011). Foodborne illness acquired in the United States--major pathogens. Emerg. Infect. Dis..

[62] McClane B., Doyle M.P. (2007). Food Microbiology: Fundamentals and Frontiers.

[63] Veshnyakova A., Piontek J., Protze J., Waziri N., Heise I., Krause G. (2012). Mechanism of Clostridium perfringens enterotoxin interaction with claudin-3/-4 protein suggests structural modifications of the toxin to target specific claudins. J. Biol. Chem..

[64] Schindelin J., Arganda-Carreras I., Frise E., Kaynig V., Longair M., Pietzsch T. (2012). Fiji: an open-source platform for biological-image analysis. Nat. Methods.

[65] Arganda-Carreras I., Fernández-González R., Muñoz-Barrutia A., Ortiz-De-Solorzano C. (2010). 3D reconstruction of histological sections: application to mammary gland tissue. Microsc. Res. Tech..

[66] Kreusel K.M., Fromm M., Schulzke J.D., Hegel U. (1991). Cl- secretion in epithelial monolayers of mucus-forming human colon cells (HT-29/B6). Am. J. Physiol..

[67] Madeira F., Pearce M., Tivey A.R.N., Basutkar P., Lee J., Edbali O. (2022). Search and sequence analysis tools services from EMBL-EBI in 2022. Nucleic Acids Res..

[68] Dereeper A., Guignon V., Blanc G., Audic S., Buffet S., Chevenet F. (2008). Phylogeny.fr: robust phylogenetic analysis for the non-specialist. Nucleic Acids Res..

[69] Notredame C., Higgins D.G., Heringa J. (2000). T-Coffee: a novel method for fast and accurate multiple sequence alignment. J. Mol. Biol..

[70] Janson G., Paiardini A. (2020). PyMod 3: a complete suite for structural bioinformatics in PyMOL. Bioinformatics.

