# Supplementary material for: Claudin-4 polymerizes after a small extracellular claudin-3-like substitution
Source: J Biol Chem. 2024 Aug 17;300(10):107693. doi: 10.1016/j.jbc.2024.107693 (PMC11490706; doi:10.1016/j.jbc.2024.107693)
Supplement: Supporting Figures [file mmc1.docx]

**Claudin-4 polymerizes after a small extracellular claudin-3-like substitution**

**Authors**

Rozemarijn E. van der Veen^1*^, Jörg Piontek^2^, Marie Bieck^1^, Arbesa Saiti^1^, Hannes Gonschior^1^, Martin Lehmann^1*^

^1^ Molecular Physiology and Cell Biology, Leibniz-Forschungsinstitut für Molekulare Pharmakologie (FMP), 13125 Berlin, Germany

^2^ Clinical Physiology/Nutritional Medicine, Department of Gastroenterology, Rheumatology and Infectious Diseases, Charité–Universitätsmedizin Berlin, Hindenburgdamm 30, 12203 Berlin, Germany

*Co-corresponding authors: [vanderveen@fmp-berlin.de](mailto:vanderveen@fmp-berlin.de) or [MLehmann@fmp-berlin.de](mailto:MLehmann@fmp-berlin.de)

**Contents**

[**Figure S1.** Sequence alignment of Cldn3, Cldn4, Cldn6, Cldn8, Cldn9 and Cldn17 demonstrates a key difference between meshwork versus non-meshwork formers. 1](#_Toc171081577)

[**Video S1.** Live-STED imaging of a SNAP-Cldn4 meshwork between two COS-7 cells. 2](#_Toc163141046)

[**Figure S2.** Colocalization of overexpressed Cldns with the TJ marker Ocln.. 2](#_Toc171081578)

[**Figure S3.** ZO1 levels vary at TJs in qKO cells expressing different Cldns, with no apparent difference in overall expression levels.. 2](#_Toc171081579)

**Supplementary figures and video**

**
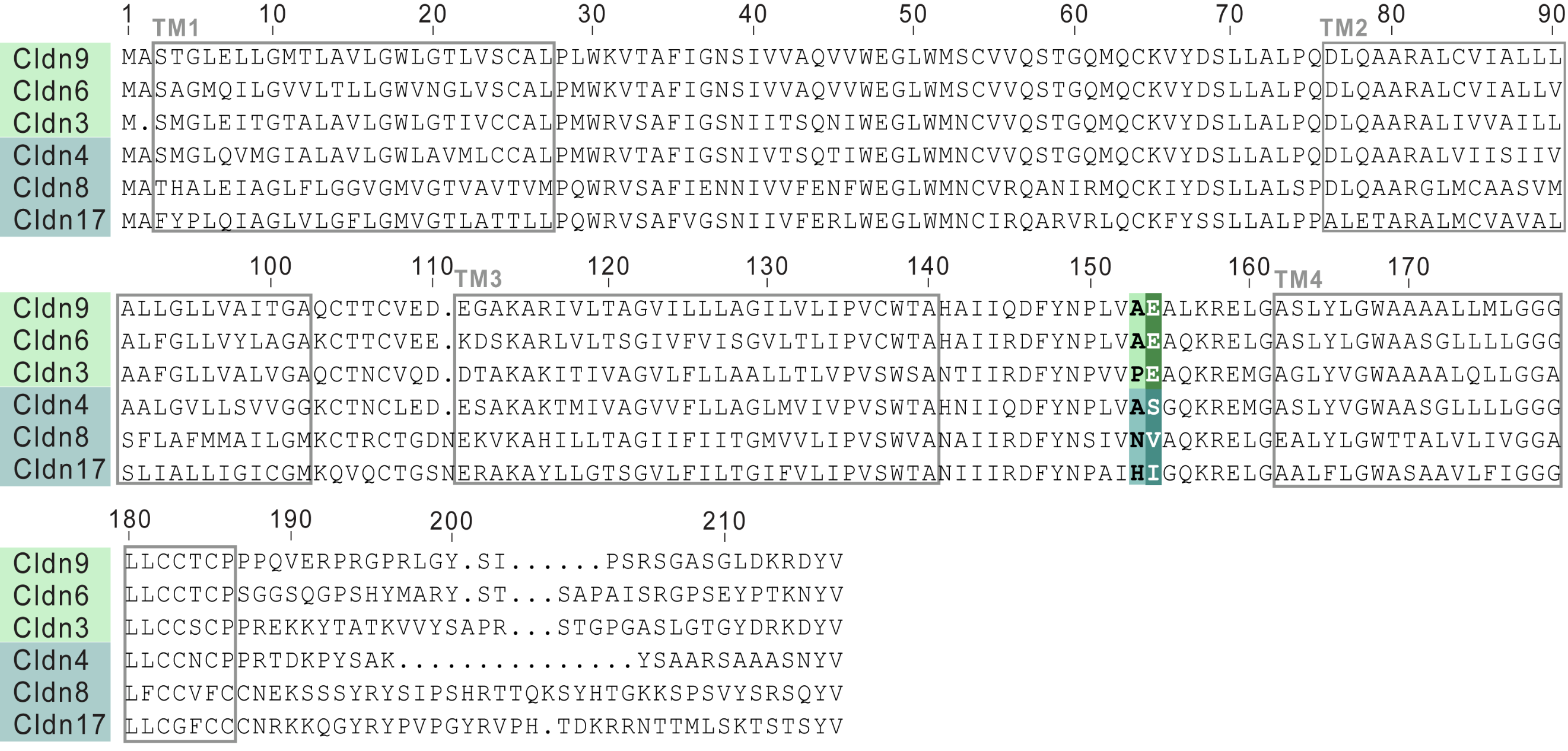
**

**Figure S1. Sequence alignment of Cldn3, Cldn4, Cldn6, Cldn8, Cldn9 and Cldn17 demonstrates a key difference between meshwork versus non-meshwork formers.** Meshwork formers are indicated in green, non-meshwork formers in blue. Human sequences were used. Numbering corresponds to Cldn9. The transmembrane helices (TMs) are indicated in grey. The key difference identified by sequence alignment (position 154 in ECS2) is highlighted in white. The neighboring residue (position 153) that was additionally mutated based on predictions from MD simulations, is highlighted as well.

**Video S1. Live-STED imaging of a SNAP-Cldn4 meshwork between two COS-7 cells.** Scale bar: 1 μm.


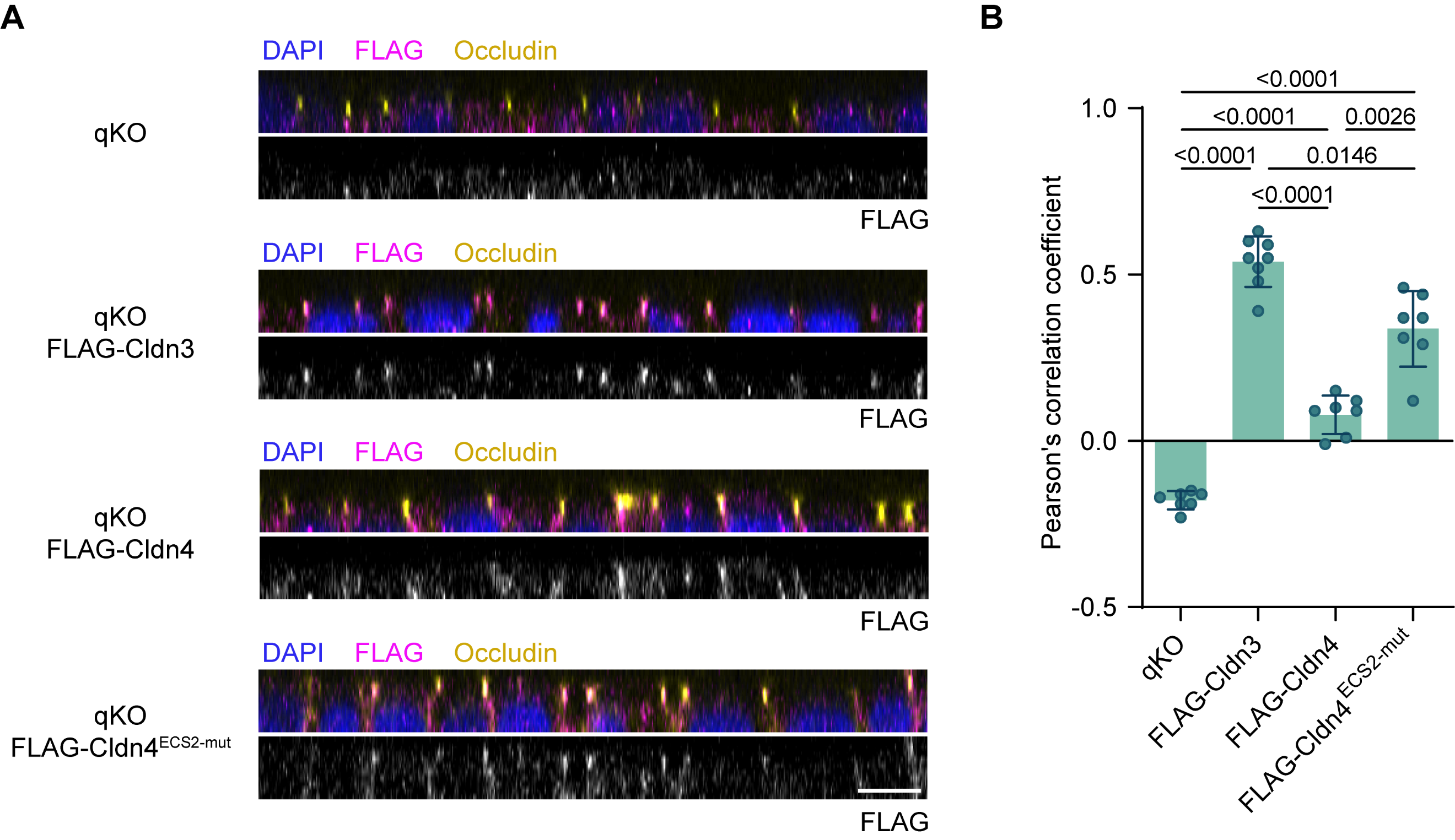
*Video is provided as a separate file.*


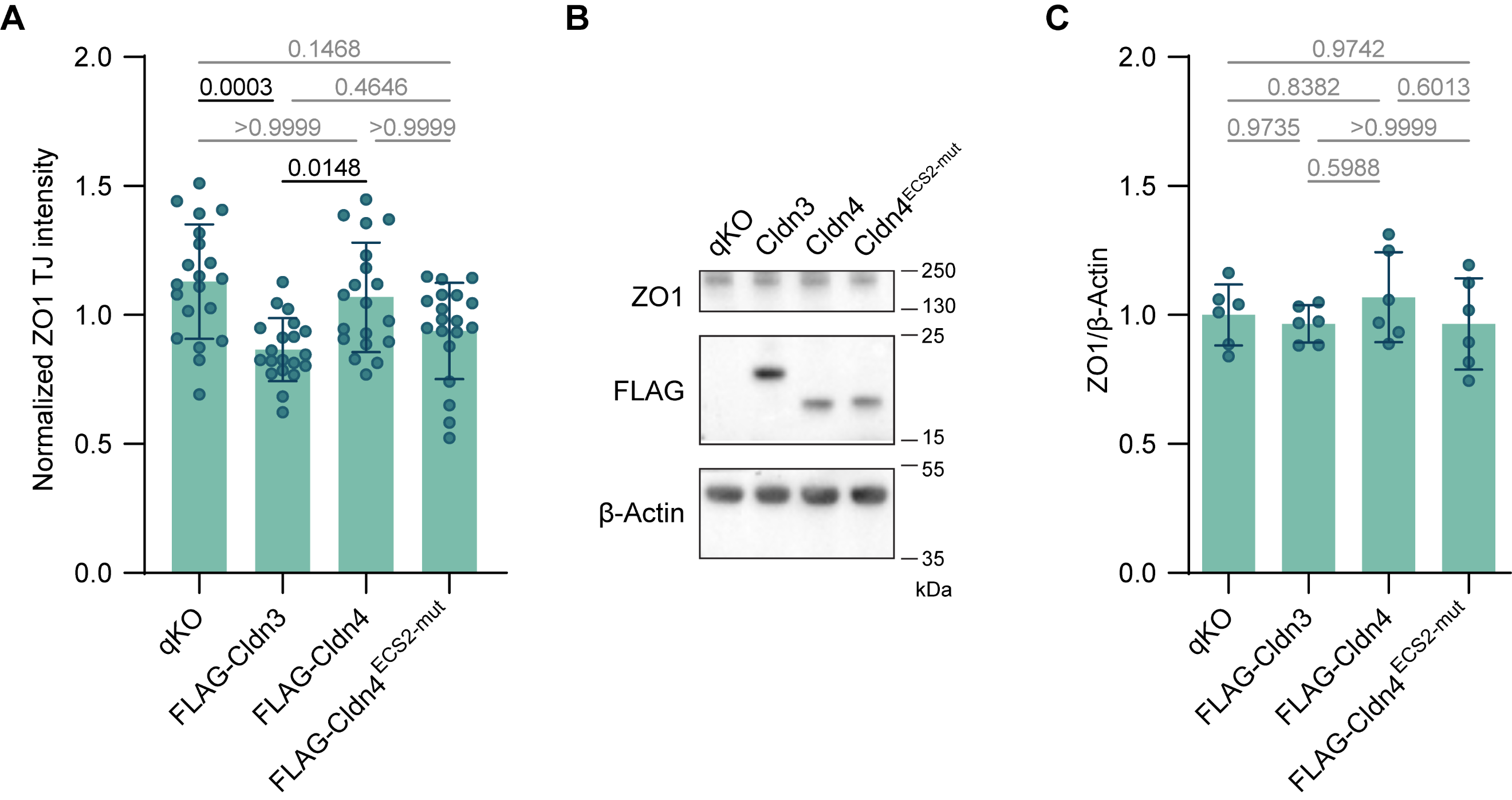
**Figure S2. Colocalization of overexpressed Cldns with the TJ marker occludin.** A, Membrane and TJ localization of FLAG-Cldn3, -Cldn4 and -Cldn4^ECS2-mut^ (magenta/grey) in qKO cells, co-stained with the nuclear marker DAPI (blue) and the TJ marker occludin (yellow). Representative orthogonal views of 10 µm z-stacks are shown. Scale bar: 10 µm. B, Colocalization between occludin and FLAG-tag in qKO cells (expressing FLAG-Cldn3, -Cldn4 and -Cldn4^ECS2-mut^). Each dot represents one 10 μm z-stack (n=7-8). Mean ± SD is shown and Brown-Forsythe and Welch’s ANOVA tests (p < 0.0001 for both) with Dunnett’s T3 multiple comparison test were performed. Adjusted p-values ≤ 0.05 are shown.

**Figure S3. ZO1 levels vary at TJs in qKO cells expressing different Cldns, with no apparent difference in overall expression levels.** A, Normalized ZO1 intensity at the TJ of qKO cells (expressing FLAG-Cldn3, -Cldn4 or -Cldn4^ECS2-mut^). Each dot corresponds to the mean ZO1 TJ intensity within one image (n=19-20, from 2 independent rounds of immunocytochemistry). Per round, the data was normalized to the average intensity of all images. Mean ± SD is shown and a Kruskal-Wallis test (p ≈ 0.0004) was performed, followed by a Dunn’s multiple comparison test. Adjusted p-values ≤ 0.05 are shown in black, adjusted p-values > 0.05 in grey. B, Overall expression of ZO1 in qKO cells (expressing FLAG-Cldn3, -Cldn4 or -Cldn4^ECS2-mut^) was assessed with immunoblotting. β-actin was used as a loading control, and FLAG-Cldn expression was demonstrated with a FLAG antibody. C, ZO1 levels in qKO cells (expressing FLAG-Cldn3, -Cldn4 or -Cldn4^ECS2-mut^) as measured from immunoblots (n=6), normalized to β-actin levels. Per immunoblot, the values were normalized to the average ZO1/β-actin of all conditions. Mean ± SD is shown and a one-way ANOVA (p = 0.5623) was performed, followed by a Tukey’s multiple comparisons tests. All adjusted p-values (are > 0.05 and) are shown in grey.
